# Mindfulness-Based Student Training Leads to a Reduction in Physiological Evaluated Stress

**DOI:** 10.3389/fpsyg.2020.00645

**Published:** 2020-05-14

**Authors:** Andreas Voss, Martin Bogdanski, Bernd Langohr, Reyk Albrecht, Mike Sandbothe

**Affiliations:** ^1^Institute of Innovative Health Technologies (IGHT), Ernst-Abbe-Hochschule Jena, Jena, Germany; ^2^Jena Achtsamkeit, Jena, Germany; ^3^Faculty of Social and Behavioral Sciences, Friedrich-Schiller-University Jena, Jena, Germany; ^4^Department of Social Work, Ernst-Abbe-Hochschule Jena, Jena, Germany

**Keywords:** mindfulness-based stress reduction, mindfulness-based interventions, autonomic regulation, heart rate variabiity, pulse wave variability

## Abstract

**Background and Objective:**

In today’s fast-paced modern lifestyle, chronic stress has become a serious issue with potential consequences for our physical and mental health. The concept of mindfulness and its derived Mindfulness-Based Stress Reduction (MBSR) program is considered to be an effective stress management technique for patients as well as for healthy persons. The effects of MBSR interventions on their participants have been subject of previous research, especially with regard to psychological or social science approaches using self-reports and questionnaires. In contrast, medical investigations in this field have been less frequent and often somehow limited, for example, addressing only absolute (discrete) mean values for heart rate or blood pressure.

**Methods:**

In this study, we have evaluated a Mindfulness Based Student Training program (MBST) by applying methods of biosignal analysis to examine its impact on the training participants’ autonomic regulation. This intervention program included classical MBSR elements but was adapted to suit the normal daily needs of university students. We obtained the electrocardiogram, finger-pulse plethysmography, and respiration activity from students participating in either the intervention group (IGR, 38 subjects) or a passive control group (CON, 35 subjects) prior to and after 8 weeks of MBST intervention.

**Results:**

When comparing various indices from heart rate variability, pulse wave variability, and respiration in linear and nonlinear domains, significant changes in the autonomic regulation were observed for the IGR group after 8 weeks of MBST.

**Conclusion:**

The results indicate a reduced stress level exclusively for the intervention participants, and therefore, we assume a health benefit from the MBST program.

## Introduction

Mindfulness and mindfulness meditation, as a medical treatment program, were first introduced by Jon Kabat-Zinn in the 1970s (Kabat-Zinn, 1982) and characterized as an awareness of the present moment and “means paying attention in a particular way: on purpose, in the present moment, and nonjudgmentally” (Kabat-Zinn, 1990; Kabat-Zinn, 1994). The concept from Kabat-Zinn was inspired by Buddhist practices and adapted for the Western world by leaving out the religious and cultural context of its origins (Kabat-Zinn, 2011). Furthermore, it was the basis for the Mindfulness-Based Stress Reduction (MBSR) program as a treatment protocol to mediate mindfulness to patients with chronic pain in the first place but, later, also to other patients as well as healthy individuals with a wide range of health conditions (Kabat-Zinn, 1990). Today, MBSR is considered to be an effective method for stress reduction and a technique for stress management in many areas of life (Frank et al., 2013; Smith, 2014; Stefan et al., 2018). The World Health Organization (WHO), for example, credited stress at the workplace as being the cause for unhealthy consequences (WHO, 2010). This kind of stress also applies to university students struggling with the demands of many courses and the taking of exams, in particular (Hamaideh, 2011; Ribeiro et al., 2018). The perception of stress and the capacity to withstand unhealthy consequences is very individual. However, if exposed to such pressure continuously or frequently without appropriate recovery periods, the perceived mental stress can become chronic and may cause or promote physical and mental diseases. This, for example, includes an impaired immune system, which could promote infections, inflammatory processes, and/or sickness behavior (Ader, 2001; Glaser and Kiecolt-Glaser, 2005; Tian et al., 2014; Wirtz and Von Kanel, 2017). Furthermore, this could also increase the risk of coronary heart- (CHD) or cardiovascular (CVD) diseases (Ghiadoni et al., 2000; Strike, 2003; Chandola et al., 2008; Schwartz et al., 2012; Wirtz and Von Kanel, 2017). Finally, chronic mental stress may also lead to anxiety disorder, depression, or burnout (Connor and Leonard, 1998; Marin et al., 2011). It can be concluded that stress reduction represents a health benefit, which would, thus, also enhance one’s quality of life. One way to achieve this may be through participation in an MBSR training. In accordance with the observed effects of mindfulness, in general, and MBSR on the mind and body, we can assume that these effects are physiologically measurable as biomarkers of the autonomic regulation (May et al., 2016; Pascoe et al., 2017). The effectiveness of MBSR has been a frequent subject of research. However, in most cases, the evaluation approach utilized methods from the fields of social science or psychology, using self-reporting surveys or questionnaires as measurement tools (Christopher et al., 2015; Hindman et al., 2015; Janssen et al., 2018; Stefan et al., 2018). Only a minor part of research on this topic reported findings that were based on a medical approach using biomarkers, for example, through investigating cytokine expressions or cortisol levels (Sanada et al., 2016, 2017). While the details (both in terms of different observed populations and the underlying processes) are still the subject of research, overall, they conclude that mindfulness interventions appear to have a positive effect on inflammatory processes, cytokine expression, and cortisol levels (the latter two were reduced). Additionally, there are studies on the effects of mindfulness interventions via measurements of clinical physiological biosignals (Abbott et al., 2014; Sharma and Rush, 2014; Wielgosz et al., 2016). The latter approach was often limited to very few aspects of MBSR effects on the biosignals and functions of the autonomic regulation; for instance, the analysis only included a single and/or non-continuously derived biosignal with a restricted number of features to describe its characteristics (e.g., only one discrete mean value, but no variability indices). In this study, we applied a specific type of stress reduction method, which has been optimized for students (Mindfulness-Based Student Training—MBST) including classical elements of MBSR interventions. This training was accompanied by measuring several medical biosignals. Based on the findings of the aforementioned literature on the positive effects of MBSR and similar mindfulness interventions, we assume that the MBST intervention will result in physiological stress reduction and health benefits for our university students. We further assume that this effect can be shown objectively by measuring respective changes in the autonomic regulation of the participants. This included analyses of electrocardiogram (ECG), finger pulse (PPG), and breathing activity (RESP), characterizing changes in heart rate variability (HRV), pulse wave variability (PWV), and RESP. This work is part of an interdisciplinary project to introduce MBST courses into the daily life of university students with the aim of enabling the participants to develop and maintain an independent daily practice of 15–20 min. Based on this integration into the daily routine, a more conscious handling of stress (resulting in a certain stress reduction) as well as improved emotional regulation and self-organization, especially at exam time, should be taught. At the moment, this publication of our explorative study only handles the medical evaluation of our MBST training in order to prove its positive effects on the students. A social science-related study (another objective of this project) is currently under investigation.

## Materials and Methods

### Ethical Approval and Informed Consent

All procedures performed in the study involving human participants were approved by the Institutional Ethics Commission of the University Hospital Jena (4509-08/15) and in accordance with the 1964 Helsinki declaration and its later amendments. Informed consent was obtained from all individual participants included in the study.

### Study Design

The study is designed as a (restricted randomized) controlled trial investigating the measurable effects of a 12-week MBST intervention for university students. All participants are measured at several specific times prior to, during (after 8 weeks of MBST), at the end, and followed-up for months after completion of the MBST intervention. Owing to the ongoing measurement process, this publication solely represents an initial assessment of our work, limited to the intervention group and the control group and based on the first two measurement points.

### Participants

The announcement for participating in the MBST course and the related evaluation study has been realized via word-of-mouth at the campus and during lectures as well as within the online lecture directory and web portals of the university. The further communication with interested students took place by email and short message service. Eligibility criteria for the participants during recruitment were being a full-time student at the university at an early semester to guarantee a long-time participation in the follow-up of the study for at least 1 year. In addition, the following subjects were excluded from the study after an initial meeting: students with known heart disease, hypertension, diabetes, pregnant women, (semi-)professional athletes, and those with advanced experience in mindfulness training. All participants were bachelor or master students at a local university and came from different departments and academic fields. The majority were students in social sciences; however, some also came from the fields of economics or business engineering. At the final stage of the recruitment, there were 80 participants assigned to two groups—the MBST intervention group (IGR) and a passive (no MBST intervention) control group (CON). The assignment was based on a chronological registration process (“first come, first serve”). The first 40 participants were assigned to the intervention group, the latter ones to the control group. After completion of the registration and right before starting the MBST intervention, 39 participants in IGR and 37 in CON successfully accomplished the adjustment measurement session (M0) and the first real data recording session (M1, pre-intervention). After 8 weeks of the intervention (measurement M2), 38 and 35 participants finished this measurement session in IGR and CON, respectively. The stepwise decrease is explained by study terminations or cancellation for private reasons. Both groups consisted primarily of female participants of nearly the same ratio. Age and body mass index (BMI) were also comparable. Such information is shown in Table 1.

**TABLE 1:** Biometric data from participants in both groups at measurement points M1 (before MBST) to M2 (after 8 weeks of MBST).

**Group**	**N (participants)**	**Sex (female/male)**	**Age (mean ± sd, years)**	**BMI (kg/m^2^)**
IGR	38	32/6	24.5 ± 3.7	22.2 ± 2.8
CON	35	31/4	24.1 ± 7.1	22.6 ± 5.0

*Note. IGR, intervention group; CON, control group.*

### MBST Intervention

The performed MBST course for the IGR group consisted of a 12-week intervention period, containing several elements of “classical” MBSR. The program was conducted in weekly sessions of 90-min each, except for one 5-h intensive practice session. The program was framed by an additional introductory and closing session, mainly for organizational purposes. The mindfulness practices and themes were being conveyed only during the 12 sessions in between. Participants were encouraged to do additional mindfulness exercises at home for about 20 min a day with the guidance of audio recordings. The experiential mindfulness practices and contentual themes taught in MBSR were partly adapted and tailored to students’ needs regarding the specific requirements of university life such as mindful exam preparation. The sessions included formal and informal exercises, including the “body scan,” mindful yoga, sitting- and walking meditation, as well as the request to keep a mindfulness diary. One main objective was to strengthen the conscious handling of stress at university as well as in private terms. Other aims were enhanced self- and emotion regulation of the participants, as well as improvements in awareness, communication skills, daily structure, and overall quality of life, in general. To maintain a small training group size and thus improve the intervention quality for all persons involved, the IGR group was organized into two training groups, each with a separate trainer. Both are certified and experienced MBSR trainers and conducted their sessions in close consultation in order to secure the use and maintenance of the same contents and procedures in each group. The sessions took place in the university auditorium, which could offer a large-scaled and brightly lighted room in a well-known location and having short, easy access for all participants during their university hours. A curriculum of the MBST sessions is attached as Supplementary Material. It gives an overview of the content of the weekly intervention sessions.

### Data Acquisition

The evaluation measurements for both groups were conducted at various times before, during, and after the MBST intervention to follow-up the subjects’ progress and to observe the changes in their autonomic regulation. In this report, we focus on the comparison of measurements M1 (directly prior to the start of MBST) and M2 (after 8 of 12 weeks of MBST) to show a first assessment of this program. This first analysis was determined after 8 weeks of MBST due to the time interval being in accordance with typical MBSR workshop durations. More findings of this evaluation study (including the measuring points M3 to M6) will be published later, when the measurements and analyses have been concluded. The entire measurement concept is shown in Table 2. The room exclusively reserved for this study was situated at the university campus. The measurements were made sequentially and were always performed by the same examiner. The participants were asked to sit on an office chair in a calm state and should preferably not move during the recordings. After the application of the sensors and checking for signal quality, the research associate obtained at least 11 min of records from the respective subject. The first minute of the record was later discarded due to adaption- and calming-down purposes for the subject, resulting in a total 10-min record for analysis. The following biosignals were obtained for each measurement: two channels of ECG, a fingertip pulse wave photoplethysmogram (PPG), and two channels of breathing activity (via respiration belts). All these biosignals are continuously and non-invasively recorded. They represent the basis for deriving the characteristics of a persons’ autonomic regulation. The two bipolar ECG channels were practically shortened equivalents to Einthoven’s lead II and III, with adhesive electrodes placed under the collar bones and at the left abdomen. The ground electrode was placed at the right abdomen. The pulse wave was derived by a standard fingertip pulse photoplethysmogram, placed at the right index finger. Finally, one respiration belt was attached to the thorax (right under the breast), and the other to the abdomen to suit the primarily thoracic or abdominal breathing of the respective subject. All biosignals were recorded via a Porti7 bioamplifier from TMSI Netherlands at a sampling frequency of 1,600 Hz and stored to a connected monitoring PC. This resulted in one file (dataset) per measurement and per subject. To ensure comparability, we measured IGR and CON in the same way and within the same course of time (regarding measurement dates). At each measurement session (M0, M1, and M2), we noted some information about the (medical) condition and behaviors of the participants, such as age, height, weight, pregnancy, smoking, (semi) professional athletes, heart disease, diabetes, hypertension, other noteworthy diseases (which could affect the autonomous body functions), current medication, and “other special remarks.” These other special remarks could include, for example, potentially important information about the participant’s current emotional state or recent physical exertion. In addition, we asked the participants at each measurement session about their current subjective stress state on a four-level scale (0 relaxed, 1 nervous, 2 stressed, 3 very stressed) to get a brief impression of their current mental state. Besides, the amount of individual mindfulness exercises at home of the intervention group was recorded for the time during and after the end of the MBST course. In this first analysis, we have not yet included all aspects of the recorded meta-information that could affect the results in any way, but we have already excluded professional athletes, pregnant women, and cases of heart disease, hypertension, or diabetes. In the ongoing study we will include more of the meta-information noted, which could lead to new insights into the influence of some of the variables. Furthermore, a detailed questionnaire was given at each measurement for completion. This contained standardized queries organized in several sections, relating to personal data, study-related life, general well-being, mindfulness, stress level, activities for stress reduction, sports activities, and life planning. Investigations regarding or including this social science part are still under investigation.

**TABLE 2:** General measurement concept of the entire study.

**Measurement**	**Description**
M0	Acclimation measurement
**M1^a^**	**Before MBST**
**M2^a^**	**After 8 weeks of MBST**
M3	After end (12 weeks) of MBST
M4	3 months after end of MBST
M5	6 months after end of MBST
M6	1 year after end of MBST

*^a^Only M1 and M2 (boldface) are subjects of this publication.*

### Pre-processing

After their acquisition, the raw data recordings had to be pre-processed in order to convert them into a utilizable shape. Every file containing the biosignals of a specific measurement was cut to a length of 10 min by discarding leading values. Then the PPG and RESP signals were bandpass-filtered with an eighth-order Butterworth with cutoff frequencies from 0.04 to 6 Hz and 0.05 to 5 Hz, respectively. Subsequently, only one ECG and one RESP channel (from formerly two in each case) were chosen for further processing by selecting the channel offering the higher signal quality. In the next step, characteristic features of the chosen signals were detected. In a first stage, an automatic peak detection (adapted and configured for each biosignal) delivered most of these points. In a second stage, the results were checked and manually edited to discard wrong or add missed peaks if necessary. The detected R-peaks from the ECG provided the RR-interval time series, representing a tachogram for later heart rate variability (HRV) analysis. Further, we obtained from the PPG all time points and amplitudes of the systolic maxima and diastolic minima, forming the PSYS and PDIA time series, respectively (also called pulse systogram and pulse diastogram, in an analogy with blood pressure data). Later, this data allowed us to analyze PWV as a kind of surrogate for blood pressure variability (BPV). To approximate typical values of blood pressure, the PSYS and PDIA time series were also linearly transformed. The linear approach enables us to avoid corruption of the contained components and relations within the time series, but to use well-established methods of our research group’s BPV analysis algorithms. Finally, the characteristic points of maxima and minima in the RESP signal provided the time series for inspiration (RESPin) and expiration (RESPex) of the breathing activity, allowing for later RESP analysis. In a last pre-processing step, we applied an adaptive filtering to the obtained RR- interval-, PSYS- and PDIA time series to correct and interpolate abnormal peaks caused by detected arrhythmic beats or technical artifacts that could distort further analysis. After this correction, the RR-interval time series was termed NN-interval time series (normal-to-normal beat interval).

### Feature Extraction

#### Standard Parameters

Based on the pre-processed time series, we performed several feature extraction methods. These methods provided information describing the characteristics of NN, PSYS, PDIA, and RESP as markers of the autonomic regulation and, thus, as markers of a distinct stress level. This process included time- and frequency domain, and nonlinear dynamics (NLD) calculations, resulting in uni- as well as bi-/multivariate parameters. The following standard parameters for heart rate variability in time- and frequency domains, as proposed by the Task Force of the European Society of Cardiology and the North American Society of Pacing and Electrophysiology, were calculated (prefixed with HRV_) (Force, 1996):

• meanNN—mean value of all NN-intervals in (ms)
• sdNN—standard deviation (sd) of all NN-intervals in (ms)
• sdaNN1—standard deviation of the averages of NN-intervals in all 1-min segments in (ms)
• RMSSD—square root of the mean squared differences of successive NN-intervals in (ms)
• pNN50—proportion derived by dividing the number of interval differences of successive NN-intervals greater than 50 ms by the total number of NN-intervals
• LF/HF—ratio of low frequency (0.04–0.15 Hz) and high frequency (0.15−0.4 Hz) power spectra estimates
• LFN—normalized LF, described by $\text{LFN}=\frac{\text{LF}}{\left(\text{LF}+\text{HF}\right)}$
• HFN—normalized HF, described by $\text{HFN}=\frac{\text{HF}}{\left(\text{LF}+\text{HF}\right)}$

According to other research efforts and clinical practices, the PPG is appropriate to obtain cardiovascular indices (Allen, 2007; Elgendi, 2012; Liang et al., 2018; Martinez et al., 2018). Besides the HRV from the ECG, it is, therefore, another approach to describe the expressions of a persons’ autonomic regulation. For pulse wave variability from the PPG, we assessed the following standard parameters to describe the systolic and diastolic amplitudes, and their variabilities in time domain (prefixed with PSYS_ and PDIA_):

• meanAmp—mean systolic maximum (PSYS) and diastolic minimum (PDIA) amplitude values
• sdAmp—standard deviation of all PSYS and PDIA values
• cvAmp—coefficient of variation of all PSYS and PDIA values

For physiological respiration, we used the following standard parameter in time domain (prefixed with RESP_):

• rateBPM—mean respiratory rate for the entire time series in (breaths per minute)

#### Additional Parameters

Besides the presented standard parameters, we further calculated sophisticated parameters, which describe the variabilities and interactions in the examined time series, particularly characterizing nonlinear interactions. These parameters include indices from correlation and transinformation (mutual information) computations that describe the statistical relationship or the dependence (more commonly, the similarity) of two variables (in our case between the time series). Both methods can be used to examine dependencies in a bivariate setting, for instance, between NN and PSYS or NN and PDIA (cross-correlation, cross-transinformation), but also in a special case to investigate the self-similarity within one time series with a time-shifted copy of itself (auto-correlation, auto-transinformation). Whereas the correlation, in our case more specifically, the Pearson correlation coefficient *r* is limited to account solely for linear relationships, the transinformation is also capable of identifying nonlinear relationships. Both can serve as a measure to describe dependencies and interactions between the different functions of the autonomic regulation (Pompe et al., 1998; Hoyer et al., 2002).

Furthermore, we used methods to quantify the complexity as an expression of randomness for the given time series. This was done by calculating different entropies of the histogram for the respective time series (NN, PSYS, and PDIA), representing a measure for diversity of its distribution. We could, therefore, estimate different variations of Rényi entropy (by varying the order α) including Shannon entropy (which is derived by the special case α = 1) (Kurths et al., 1995).

Using methods of symbolic dynamics (SymDyn), we computed word probabilities to further describe the time series and their variability (applies for HRV and PWV). These words consist of symbols from a defined set of characters—in this case, three successively occurring symbols per word from an alphabet of A_SymDyn_ = {0, 1, 2, 3} and address for the relation of each variation within the time series and determine their probability. This results in word probabilities for all possible patterns (pW000, pW001…pW333) of the variation sequences. Subsequently, we derived the summarized indices wpsum02 and wpsum13, which provide the proportion of words only consisting of the symbols “0” and “2,” as a measure for decreased variability, or “1” and “3,” as a measure for increased variability, respectively (Kurths et al., 1995; Voss et al., 1996).

Another approach we used to analyze nonlinear interactions between NN- and PSYS/PDIA-time series were methods of (bivariate) high-resolution joint symbolic dynamics (HRJSD) and multivariate HRJSD (mHRJSD). Similar to the classical symbolic dynamics described above, HRJSD describes variations in the time series via words of symbols. In this case, symbolic dynamics are not only analyzed within one time series but also for the interaction between two or more time series simultaneously, which means to illustrate the occurrence of different patterns of variations within different time series (for example, NN, PSYS, and PDIA) at the same time, as well as their probabilities. In this matter, one word consists of three symbols from the alphabet A_HRJSD_ = {0, 1, 2,}—with “0” representing a decrease, “1” no change, and “2” an increase. This results in typical word families to describe the behavior of the given signal. For instance, the pattern E0 (“000”) for three successive decreases indicating a great drop but no variation, the pattern LU1 (“122” as one example from several in this type) for a slight increase with one variation, or the pattern V (“012” to mention one example for this type) showing three variations within one word. This way, the proportions and probabilities of variability indices, based on slight, great, or no variation patterns, can be estimated, even for two or more time series (Schulz et al., 2013).

Finally, we performed segmented Poincaré plot analysis (SPPA) for the NN-, PSYS-, and PDIA-time series to further calculate variability indices. Besides the traditional parameters of Poincaré plot analysis (PPA)—like SD1, SD2, and their ratio—this extended method adds more options to describe the point cloud in the scatter plot, derived from plotting one data point against the succeeding data point. The whole plot around the focus of the point cloud is split into rectangles that segment the diagram and allow for the addressing of rows and columns (of segments) with valued occurrences of points. These result in intrinsic probabilities to describe the shape of the point cloud more specifically than does the classical approach. This power of additional variability indices via SPPA may lead to new insights which PPA alone fails to provide (Voss et al., 2010).

### Statistical Analysis

The calculated parameters were statistically analyzed to identify significant changes from M1 (prior to MBST) to M2 (after 8 weeks of MBST) in both IGR and CON. This question followed a paired data setup that compares parameters from IGR in M1 and M2, which also applied to CON. Since the observations, i.e., the received parameters per group and measurement, did not reliably match the assumptions of a parametric test (like normal distribution for a paired *t*-test), we instead used non-parametric tests for our paired data. This analysis was done automatically via a MATLAB script that calculates the test for significance, via either Wilcoxon signed-rank test or sign test. In a first stage, every pair of tested variables was checked for whether they come from the same distribution or not. This was performed due to the test assumptions of symmetry and that the observations come from the same population—meaning equal distributions of the compared observations. If the two-sample Kolmogorov–Smirnov test confirmed the same distribution for a tested pair, then the Wilcoxon signed rank was used in the second stage; otherwise, the sign test was used. Based on the obtained results, we were able to check for statistically significant changes for the intervention group within the first 8 weeks of MBST training and could also examine if these changes applied for the control group as well (during the same time period). Furthermore, we conducted a Mann–Whitney *U*-test to examine possible starting differences between both groups at M1. Again, we used a non-parametric test that does not require the rigorous assumptions of parametric tests. All statistical tests were carried out at the significance levels α_1_ = 0.05, α_2_ = 0.01, and α_3_ = 0.001 to account for significant, moderate significant, and highly significant parameters, respectively. For more condensed results, we finally tested all significant parameters for their cross-correlation. In this way, we could focus and summarize the statements within the different methodical families and discard some highly correlated parameters (threshold *r* > 0.8) that offer the same key message but no new knowledge. In addition to the calculated significances, we computed the respective effect sizes of the comparisons. Since non-parametric tests were used in the statistics, which also produced a z-value, we have calculated the effect size according to:

$$r=\frac{z}{\sqrt{N}}.$$

We also checked these results with the more general and robust method of Cliff’s Delta with:

$$\Delta =\frac{\left(\sum x_{1}>x_{2}\right)-\left(\sum x_{1}<x_{2}\right)}{n_{1}\,n_{2}}.$$

## Results

We investigated changes in the autonomic regulation for the IGR group between M1 (prior to MBST) and M2 (after 8 weeks of MBST) in relation to the CON group. In the end, the measurements revealed significantly changed parameters in IGR, in contrast to CON (see within-group results in Table 3). On the one hand, there were nearly no significant changes regarding the most common standard parameters from time and frequency domains. In this context, the assessed RESP and HRV parameters were not significantly changed within IGR. Only three standard parameters, which are related to the variability in PSYS and PDIA, showed a significantly decreased value. These were the standard deviations of PSYS and PDIA (with *p* < 0.015 for PSYS_sdAmp, *p* < 0.011 for PDIA_sdAmp), as well as the coefficient of variation in PSYS (*p* < 0.003 for PSYS_cvAmp). On the other hand, by considering the additional information content from NLD and SymDyn methods, several parameters unveiled significantly changed values within IGR. A decreased PSYS_renyi2 (*p* < 0.001) and an increased PSYS_wpsum02 (*p* < 0.001) at M2 indicate a lower complexity in PSYS and, therefore, a lower pulse wave variability after 8 weeks of MBST training. Also, the SPPA for PSYS showed significant alterations with a decrease in column five (*p* < 0.006) and an increase in column seven (*p* < 0.001). In addition, the slope of the auto-transinformation for PSYS (*p* < 0.007 for COR&TI_a31PSYS) and the amplitude of the cross-correlation maximum between PSYS and PDIA (*p* < 0.003 for COR&TI_ymaxPSYSPDIAcor) were significantly reduced at M2. Finally, the (m)HRJSD revealed significant changes in the occurrence of specific single patterns as well as the interaction of specific patterns (with *p* < 0.006 for PSYS-LU1, *p* < 0.001 for PDIA-V, *p* < 0.005 for PDIA-E0/NN-LU1, *p* < 0.009 for PDIA-E0/NN-LA1, *p* < 0.002 for NN-E0/PSYS-E0/PDIA-E0, *p* < 0.001 for NN-LU1/PSYS-E2/PDIA-E2, *p* < 0.008 for NN-V/PSYS-LD1/PDIA-LA1, and *p* < 0.003 for NN-V/PSYS-V/PDIA-LA1).

**TABLE 3:** Within-group results for IGR and CON at M1 and M2 (means and standard deviations; Wilcoxon signed-rank test and sign test for statistical significance, effect size *r*).

	**IGR**		**CON**	
**Parameter**	**M1 Mean ± sd**	**M2 Mean ± sd**	**M1 Mean ± sd**	**M2 Mean ± sd**
**RESP_**				
rateBPM	13.33.4	13.33.4	12.73.4	13.33.4*†
**HRV_**				
meanNN	792.57118.19	786.88104.89	795.04101.33	762.74135.75†
sdNN	52.1819.67	49.317.52†	50.3716.61	47.8619.64†
sdaNN1	21.8710.81	21.5510.1	20.3410.05	20.698.26
rmssd	37.2518.02	34.1214.48†	35.0613.01	31.2415.98†
pNN50	0.180.17	0.150.14†	0.170.13	0.130.14†
LF/HF	3.624.72	2.323.02†	4.137.19	3.915.73
LFN	0.620.22	0.580.18	0.570.24	0.630.2†
HFN	0.380.22	0.420.18	0.430.24	0.370.2†
**PSYS_**				
meanAmp	136.816.90	138.695.94†	138.0110.86	141.2211.08†
sdAmp	7.912.93	6.592.74*†	7.694.13	7.23.94†
cvAmp	0.0580.022	0.0480.02**††	0.0550.027	0.0510.027†
renyi2	2.310.54	2.010.5***††	2.250.7	2.140.72†
wpsum02	0.50.24	0.640.2***††	0.520.26	0.560.29†
SPPA_col5	11.573.48	9.292.91**††	12.252.35	11.642.42†
SPPA_col7	37.716.51	41.835.91***††	35.824.81	36.734.71
**PDIA_**				
meanAmp	100.497.78	98.877.16†	98.8612.43	97.4411.04
sdAmp	8.403.12	7.252.61*†	8.514.81	8.234.50
**COR&TI_**				
a31PSYS	−1.2880.133	−1.2060.134**††	−1.2890.092	−1.2770.116
ymaxPSYSPDIAcor	0.7240.132	0.6420.113**††	0.7030.134	0.6670.127†
**HRJSD_**				
PSYS-LU1	0.2900.047	0.3130.033**††	0.2970.051	0.3050.05†
PDIA-V	0.0660.025	0.0780.028***††	0.0670.027	0.0670.025
PDIA-E0/NN-LU1	0.0480.019	0.040.019**††	0.0390.023	0.0370.019†
PDIA-E0/NN-LA1	0.0070.008	0.0050.005**††	0.0040.006	0.0060.007
**mHRJSD_**				
NN-E0/PSYS-E0/PDIA-E0	0.01530.0307	0.00790.0149**††	0.01620.0297	0.01610.0318
NN-LU1/PSYS-E2/PDIA-E2	0.02290.0165	0.01560.0146***††	0.02290.0207	0.02140.0192
NN-V/PSYS-LD1/PDIA-LA1	0.00120.0017	0.00230.003**††	0.00120.0019	0.00140.0028
NN-V/PSYS-V/PDIA-LA1	0.00060.0014	0.00190.0033**††	0.00110.0016	0.00070.0014†

*p ≤ 0.05; **p ≤ 0.01; ***p ≤ 0.001. †*r ≥* 0.1 (small); ††*r ≥* 0.3 (medium); †††*r ≥* 0.5 (large). The interpretation of the effect size follows the recommendation of Cohen (1988). M1, prior to MBST; M2, after 8 weeks of MBST; SPPA, Segmented Poincaré Plot Analysis; COR&TI, Correlation and Transinformation Analysis; (m)HRJSD,(multivariate) High Resolution Joint Symbolic Dynamics Analysis. Parameter descriptions in bold represent different grouping categories of variables.

**FIGURE 1:**
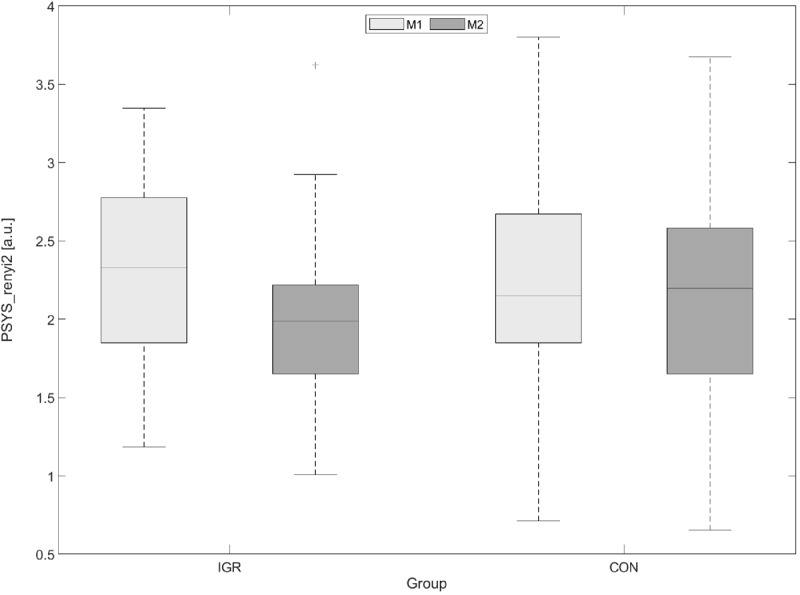
Example boxplot of PSYS_renyi2 as indicator for decreased PWV in IGR after 8 weeks of MBST and comparison to CON (left: PSYS_renyi2 significantly decreased in IGR; right: PSYS_renyi2 non-significantly changed in CON; PWV, pulse wave variability; PSYS_renyi2, the Rényi entropy with α = 2 as a quantification of the complexity in systolic pulse time series (PSYS); IGR, intervention group; CON, control group; MBST, Mindfulness Based Student Training; M1, prior to MBST; M2, after 8 weeks of MBST).

These respective significant alterations from M1 to M2 did not occur within CON, indicating exclusive changes in IGR. Particularly noticeable is that PSYS and PDIA activities and interactions seemed to be the key driver for significant changes in this MBST investigation, unlike for HRV or RESP. Based on the outcome of our measurements and analysis, it can be concluded that the conducted first 8 weeks of MBST training provoked an impact on the student participants that is measurable via indicators of the autonomic regulation. An exemplary presentation of a highly significantly changed parameter in IGR, representing the reduced PWV in contrast to CON, is shown in Figure 1. The significant changes shown are supported by the respective effect size, most of which indicate medium changes. Besides, the additional verification using Cliff’s Delta resulted in similar findings (not reported in the tables).

The direct comparison between IGR and CON at M1 indicated practically no difference (except for HRJSD_PDIA-E0/NN-LA1 with *p* < 0.02), which means that both groups were coming from the same population (prior to the MBST training) and, therefore, had similar starting conditions. This is also supported by the corresponding effect size, which shows almost no changes. The respective values from the between-groups results before the MBST training are shown in Table 4.

**TABLE 4:** Between-group results for IGR and CON at M1—prior to MBST training (means and standard deviations; Mann–Whitney *U*-test/Wilcoxon rank-sum test for statistical significance, effect size *r*).

	**M1**	
**Parameter**	**CON Mean ± sd**	**IGR Mean ± sd**
**RESP_**		
rateBPM	12.73.4	13.33.4†
**HRV_**		
meanNN	795.04101.33	792.57118.19
sdNN	50.3716.61	52.1819.67
sdaNN1	20.3410.05	21.8710.81
rmssd	35.0613.01	37.2518.02
pNN50	0.170.13	0.180.17
LF/HF	4.137.19	3.624.72†
LFN	0.570.24	0.620.22†
HFN	0.430.24	0.380.22†
**PSYS_**		
meanAmp	138.0110.86	136.816.90
sdAmp	7.694.13	7.912.93
cvAmp	0.050.03	0.060.02†
renyi2	2.250.70	2.310.54
wpsum02	0.520.26	0.500.24
SPPA_col5	12.252.35	11.573.48
SPPA_col7	35.824.81	37.716.51†
**PDIA_**		
meanAmp	98.8612.43	100.497.78
sdAmp	8.514.81	8.403.12
**COR&TI_**		
a31PSYS	−1.2890.092	−1.2880.133
ymaxPSYSPDIAcor	0.7030.134	0.7240.132
**HRJSD_**		
PSYS-LU1	0.2970.051	0.2900.047
PDIA-V	0.0390.023	0.0480.019
PDIA-E0/NN-LU1	0.0040.006	0.0070.008†
PDIA-E0/NN-LA1	0.0670.027	0.0660.025*†
**mHRJSD_**		
NN-E0/PSYS-E0/PDIA-E0	0.01620.0297	0.01530.0307
NN-LU1/PSYS-E2/PDIA-E2	0.02290.0207	0.02290.0165
NN-V/PSYS-LD1/PDIA-LA1	0.00120.0019	0.00120.0017
NN-V/PSYS-V/PDIA-LA1	0.00110.0016	0.00060.0014†

*p ≤ 0.05; **p ≤ 0.01; ***p ≤ 0.001. †*r ≥* 0.1 (small); ††*r ≥* 0.3 (medium); †††*r ≥* 0.5 (large). The interpretation of the effect size follows the recommendation of Cohen (1988). M1, before MBST; SPPA, Segmented Poincaré Plot Analysis; COR&TI, Correlation and Transinformation Analysis; (m)HRJSD, (multivariate) High Resolution Joint Symbolic Dynamics Analysis.

## Discussion

In this explorative study, we investigated the impact of a student-tailored mindfulness-based intervention on the autonomic regulation of its participants. This MBST program, adapted from MBSR for the demands of daily life at the university, was accompanied by several measurements of ECG, PPG, and breathing activity obtained from subjects in the intervention group and a (non-intervention) passive control group. The comparison of the calculated variability indices of the NN, PSYS, PDIA, and RESP time series unveiled significant changes in IGR after 8 weeks of MBST intervention, which did not apply for CON.

As a result, we could show that the MBST training considerably influenced the autonomic regulation in a positive sense regarding a stress reduction, reflected by effects measured by analyzing the underlying biosignals and their derived variables. Several parameters changed significantly within IGR, whereas these same parameters within CON remained unchanged. In detail, the standard parameters describing the variabilities in heart rate, pulse wave, or breathing activity in linear functions and interactions seem to be less appropriate in demonstrating the effectiveness of the MBST training. Parameters from NLD, which quantify the degree of complexity and represent nonlinear interactions in the autonomic regulation, primarily appear to be good indicators for the training’s effectiveness. In addition, the variabilities of PSYS and PDIA, representing the PWV derived from the finger PPG as a kind of substitute for the systolic and diastolic BPV, seem to show cardiovascular activities as the key drivers behind the observed effects, since the HRV revealed no significant changes. Furthermore, if we take a detailed look at the directions of the altered values in IGR, we can conclude that the changed parameters behaved as expected. At rest, a healthy person usually should show a relatively high HRV, but a low BPV, indicating a good cardiovascular interaction and responsivity in comparison to the reduced HRV and/or raised BPV in several (cardiological and non-cardiological) diseases (Force, 1996; Rajendra Acharya et al., 2006). On the one hand, a decreased HRV and/or increased BPV (as well as PWV) may point to a stressful condition under a heavy physical or mental load, like in doing sports or taking exams. On the other hand, this could otherwise indicate a dysfunction in autonomic regulation that is associated with a higher risk for cardiovascular diseases and mortality, or mental disorders. Several studies investigated the relationship of different diseases with changes in HRV (Sgoifo et al., 2015; Bassett, 2016; Lennartsson et al., 2016; Kubota et al., 2017), as well as with changes in BPV (Sung et al., 2014; Parati et al., 2015; Gosmanova et al., 2016). Since all participants of this study were healthy subjects, we can only assume that the first explanation for any significant changes in autonomic regulation is applicable: the overall stress level and mental load (as the measurements were conducted while sitting and at rest). However, the implication of possible diseases indicated by changes in HRV, BPV, and/or PWV should demonstrate the potential value and importance of effective methods for stress reduction as a kind of health provision.

In this respect—changes in variability indices related to the autonomic regulation within IGR from M1 to M2—our results are consistent. All single changes in relevant parameters confirm each other in their imparted statement. The standard deviations of PSYS and PDIA, as well as the coefficient of variation of PSYS, were decreased in IGR at M2. In addition, the Rényi entropy, as a scale of the complexity in PSYS, also decreased. The derived probability of occurrence of words, representing a measurement for decreased variability (PSYS_wpsum02) in PSYS, increased. These univariate alterations indicate a decreased overall PWV within IGR at M2, compared to M1. This impression is enforced by the results of several bi- or multivariate parameters. The slope of the auto-transinformation of the systolic pulse time series (COR&TI_a31PSYS) was decreased at M2, which implies a higher auto-transinformation and, thus, a decreased PWV. This also points to a high nonlinear proportion, since the slope of the respective auto-correlation (COR&TI_a31PSYScor with *p* > 0.55) as a pure linear index was not significantly changed. The interaction between PSYS and PDIA estimated by the maximum cross-correlation of both time series (COR&TI_ymaxPSYSPDIAcor) was significantly decreased. Regarding the HRJSD (single) patterns, PSYS-LU1 and PDIA-V had a higher probability of occurrence at M2, resulting in a higher proportion of slight variations, as opposed to strong variations in the PSYS and PDIA time series. The remaining (m)HRJSD parameters support these findings as well. The aforementioned (single) PSYS and PDIA patterns are involved in many bi- and multivariate interactions that show significantly decreased strong variations and/or increased slight variations. In these cases, the involved NN patterns from the HRV seem to contribute randomly because their respective single patterns were not significant. This would also encourage the impression of systolic and diastolic behavioral patterns as being the key drivers of the examined processes. Finally, the two significant PSYS–SPPA parameters match our insights: the very central column seven changed to a higher occupation, while the less central column five changed to a lower probability of occurrence. This represents a more compressed and less dispersed data cloud in the segmented Poincaré plot in IGR at M2. This again, leads to the outcome of a reduced variability (Voss et al., 2010) of the pulse wave time series. Since variability indices only represent relative changes in the respective biosignal, the use of, and conclusions from, the PWV as a kind of surrogate for the BPV is appropriate. Nevertheless, blood pressure and PPG should not be confused or equated as the absolute amplitude values of both signals show different qualities. While blood pressure stands for the arterial pressure (measured in mmHg), the PPG depicts the light intensity measured in a photosensor (which is inversely proportional to the arterial blood volume variations). In the end, our results only provide significant changes in variability indices and no changes in the mean absolute amplitude values of PSYS and PDIA.

To summarize, these findings indicate a reduced systolic and diastolic variability in the pulse wave time series and, therefore, imply a lower stress level and better regulation of the autonomic body functions exclusively for the MBST intervention group after 8 weeks of training. In line with these results, we can reasonably assume that effects of the MBST program are not only measurable but also suggest a positive impact on participants’ health.

It has been difficult to compare our results to those of other studies, as the effects of MBSR on HRV, BPV, or PWV are subjects of a limited amount of publications. In addition, the conditions and methods are often inconsistent and, therefore, only slightly comparable. However, Nijjar et al. (2014) found no significant changes in HRV (mean heart rate, total power spectra, LF, HF, LFN, and HFN) between the measurements taken before and after an MBSR intervention (measured during spontaneous breathing). Ditto et al. (2006) also reported no significant changes in the mean heart rate between pre- and post-intervention (before and after a body scan meditation as part of an MBSR program), as well as no significant changes in mean systolic and diastolic blood pressure levels—neither immediately during meditation, nor between the measurement sessions during a 1-month period. They only found evidence of a significantly decreased mean heart rate directly during meditation (intervention group) and during quiet sitting (control group), compared to baseline measurements taken before the intervention. Nyklíček et al. (2013) received no significant changes for mean heart rate and HRV (in time- and frequency domains) from pre- to post-intervention baseline measures. However, they could show a stronger decrease in mean blood pressure levels for the MBSR group than for the control group after the intervention (measured at baseline and during an acute stress task). In terms of HRV, these findings support our results regarding the respective indices, since we also found no evidence of a changed HRV in IGR. The uncertainty regarding the effect of MBSR interventions on the blood pressure seems to show a more differentiated picture. This may be due to the different methods and approaches used to derive blood pressure indices such as investigating only mean levels (or single discrete values) instead of including variability indices obtained from a continuously derived signal. Like some of the above-mentioned studies, we found no changes in the mean amplitudes of systolic and diastolic pulse wave time series, but beyond that, did notice significant changes in the variability of PSYS and PDIA. Concerning the breathing activity in another study, Wielgosz et al. (2016) observed the respiration rate over a long period of time (several months) to examine the impact of a long-term mindfulness meditation program. They showed that the baseline respiration rate was slower for the intervention group compared to the controls. This cannot yet be confirmed based on our results. However, based on their results from various breathing activity measures over months, including non-significant changes from a full-day meditation session, they hypothesized a more long-range, rather than an immediate, effect on the respiration rate from the mindfulness intervention. As we can only compare the first two measurements of our study up to date, we will address possible long-term effects in the future upon the conclusion of this entire study.

Even though these preliminary results provide a general insight into the expected effects of MBST on functions of the autonomic regulation among university students, several limitations of this study are worth to be noted. First, all participants in this study were university students. Therefore, our findings cannot offer conclusions for the general public. Furthermore, the gender ratio was noticeably tilted toward females and thus imbalanced, which may yield predominant influences from specifically female factors regarding the effects of a mindfulness-based intervention (if any). The fact that most of the participating students came from the field of social sciences could reinforce a possible influence from imbalanced affinities or sensibilities for mindfulness interventions and should thus be considered with similar reserve. Although this study is not a purely randomized study, it can be assumed that the selection process of the subjects with the randomness of the registration (time presence, knowledge of the study…) was an almost randomized procedure. In addition, the current evaluation is not yet controlled for certain protocoled meta-information such as age groups, gender, BMI, quantity of sports activities (professional athletes were already excluded), amount of prior experience in meditation or yoga (those experienced in MBSR were already excluded), as well as the amount of independent mindfulness practice at home. It should therefore be noted that some of this omitted information could affect the results.

From our present results, we can conclude that the performed MBST intervention program, with MBSR elements adapted to the special needs of a university student, indeed revealed a measurable impact on the participants’ autonomic regulation. We were able to show significant changes after 8 weeks of MBST within the intervention group that did not occur within the control group. To be precise, there was no evidence of changes in the mean heart rate or HRV indices. Additionally, the effect on mean levels of systolic and diastolic pulse wave amplitudes (as surrogates for the respective blood pressures) remain somewhat uncertain, since there were no significant changes in these as well. On the contrary to the absolute mean values in PSYS and PDIA, their related variability indices (with PWV as an expression of BPV) unveiled several significant changes in the functions of the autonomic regulation within IGR. Based on our findings, we suspect that the MBST-provoked mechanisms imply more nonlinear interactions involved in the processes and possibly less affected (linear) clinical standard indices. Finally, the obtained results allow the assumption of a positive impact on the health of the MBST participants.

In the subsequent process of this study, we will investigate the long-term impact over various measurement times up to several months after the end of the MBST program. Once the data acquisition is completed, we can also control for some meta-information concerning specific sub-groups or possible classifications regarding age, sex, BMI, sports activities, amount of home practice, and more. This would enable us to examine interactions and dependencies of various factors from these biometrical data on the outcome of the biosignal indices. Besides the mere medical evaluation of the MBST intervention, we plan to combine our results with the ones from the social evaluation conducted in parallel. This interdisciplinary analysis affecting the same subjects may provide new insights.

For future research on this topic, in general, we suggest that more attention is given to a balanced gender mix, different medical conditions (excluding all or including certain ones for specific examinations), and the participation rate (applying to the attendance of the official weekly session and to the amount of home practice). Furthermore, we recommend that more nonlinear analysis methods of the obtained biosignals are addressed and to perhaps include an active, in addition to a passive, control group. Another suggestion might be to support the MBST intervention using supplementary special media guidance (audio or video) for home practice to remind and motivate participants to perform their mindfulness exercises at home.

## Data Availability Statement

The datasets generated for this study will not be made publicly available because the study has not yet been completed, further evaluations are currently in progress. Requests to access the datasets should be directed to the corresponding author.

## Ethics Statement

The studies involving human participants were reviewed and approved by the Institutional Ethics Commission of the University Hospital Jena (4509-08/15). The patients/participants provided their written informed consent to participate in this study.

## Author Contributions

AV, MB, and MS conceived and designed the experiments. BL, MB, and RA performed the experiments. AV and MB analyzed the data and contributed reagents, materials, and analysis tools, and wrote the manuscript. All authors reviewed the manuscript.

## Conflict of Interest

BL was employed by company Jena Achtsamkeit and contributed as one of the certified MBSR trainers.

The remaining authors declare that the research was conducted in the absence of any commercial or financial relationships that could be construed as a potential conflict of interest.

## Supplementary Material

TABLE S1 Overview of the MBST curriculum for the weekly intervention sessions.
