# Supplementary material for: Mindfulness-Based Student Training Leads to a Reduction in Physiological Evaluated Stress
Source: Front Psychol. 2020 May 14;11:645. doi: 10.3389/fpsyg.2020.00645 (PMC7240125; doi:10.3389/fpsyg.2020.00645)
Supplement: TABLE S1 — Overview of the MBST curriculum for the weekly intervention sessions. [file Table_1.DOCX]

Supplementary Material

Supplementary Table 1 Overview of the MBST curriculum

| **Week** | **Duration (minutes)** | **Content overview** |
| --- | --- | --- |
| 1 | 90 | **Topic Introduction to Mindfulness**  Group rules, introduction round (beginning of the experience-based mindfulness training)  Introduction of the Body Scan  Introduction of Mindfulness Diary |
| 2 | 90 | **Topic Mindfulness & Autopilot**  Body Scan with reflection  Reflection of the home exercise practice |
| 3 | 90 | **Topic Mindfulness in daily life**  Introduction of the sitting meditation  Raisin exercise |
| 4 | 90 | **Topic How we perceive the world**  Introduction of the breathing space exercise  Introduction of walking meditation  Sitting meditation with reflection |
| 5 | 90 | **Topic Dealing with limits and the own body**  Introduction of Mindful Yoga  Exchange of experience for pleasant moments in daily life |
| 6 | 90 | **Topic Stress Reasons and Stress Reaction**  Mindful Yoga  Reflection of stress in the own daily study life  Impulse presentation and discussion on stressors and stress reaction |
| 7 | 90 | **Topic Stress through assessment, stress and mindfulness**  Sitting meditation with perception of thoughts  Impulse presentation and discussion on stress and assessments |
| 8 | 300 | **Mini retreat / an intensive unit in silence (5 hours)**  Various mindfulness exercises  Mindful eating of a meal |
| 9 | 90 | **Topic Mindful Communication**  sitting meditation in silence  Partner exercise on difficult communication  Group exercise on different communication styles |
| 10 | 90 | **Topic Mindful exam preparation**  Body Scan, walking meditation  Reflection of own study practice and exam preparation  Development of supporting learning habits, presentation of results |
| 11 | 90 | **Topic Taking good care of yourself**  Yoga practice with focus on self-care  Meditation for self-compassion  Reflection of daily and consumer habits incl. digital media |
| 12 | 90 | **Topic Review and Outlook**  Meditation  Exchange for the continuation of the own exercise practice  Closing round (end of the experience-based mindfulness training) |
